# Seed Treatment with Biostimulants Extracted from Weeping Willow (*Salix babylonica*) Enhances Early Maize Growth

**DOI:** 10.3390/plants10071449

**Published:** 2021-07-15

**Authors:** Hande Mutlu-Durak, Bahar Yildiz Kutman

**Affiliations:** 1 Institute of Biotechnology, Gebze Technical University, Gebze 41400, Kocaeli, Turkey; handemutlu@gtu.edu.tr; 2 Original Bio-Economy Resources Center of Excellence (OBEK), Gebze 41400, Kocaeli, Turkey

**Keywords:** biostimulant, salinity stress, seed treatment, willow extract, salicylic acid, maize

## Abstract

Biostimulants can be used as innovative and promising agents to address current needs of sustainable agriculture. Weeping willow tree (*Salix babylonica*) extracts are rich in many bioactive compounds, including, but not limited, to salicylates and phenolics. In this study, the potential of willow bark (WB) and willow leaf (WL) extracts is evaluated as plant-based biostimulants to improve the early growth of maize (*Zea mays*) under control and salinity stress conditions. In 3 days, seed treatment with salicylic acid and willow extract increased the shoot FW of maize seedlings 130% and 225%, respectively. The root area was, on average, enhanced by 43% with SA and 87% with willow extract applications. Moreover, these extracts increased the leaf protein concentration and reduced the negative effects of salinity during early growth. Reductions in lipid peroxidation and specific activities of antioxidative enzymes by seed treatments with willow extracts suggests a mitigation of salinity-induced oxidative stress. For most reported traits, WL applications were at least as effective as WB applications. Results indicate that aqueous extracts of weeping willow leaves, as well as bark, can be used as seed treatment agents with biostimulant activity to improve seedling growth and establishment under control and stress conditions.

## 1. Introduction

By 2050, food production must be approximately doubled in developing countries and increased by 70% globally to meet the demand of an increasing population [1]. Therefore, it will become even more important to achieve maximum crop yield from the unit area in the near future [2,3]. The decreasing arable land, climatic changes and excessive usage of chemical fertilizer threaten global food safety and security. As such, sustainable, eco-friendly and also efficient agriculture practices are required for feeding the growing population [4].

In recent years, plant biostimulants, which can be a promising solution as a safe, innovative and productive approach for sustainable agricultural production, are partially reducing the usage of chemical alternatives [5,6,7]. Biostimulants can be classified in many different categories including inorganic compounds, humic and fulvic acids, seaweed extracts, protein hydrolysates, beneficial fungi and bacteria. Novel compounds with biostimulant activities are commonly characterized and added to the literature [8]. Plant-based biostimulants, which are also known as botanicals, are plant extracts or substances obtained from plants which can also be used as food additives or other products that are manufactured by pharmaceutical and cosmetic industries [9,10]. Botanical extracts include several important natural bioactive molecules, including natural phenolics [11]. These bioactive substances can increase yield and fruit quality, enhance photosynthesis, carbohydrate levels, nodule development and also improve secondary metabolite production [12,13,14]. These plant-based biostimulants can be applied to economically important plants by seed or soil treatments, as well as foliar applications both in the presence or absence of a stress condition.

Salinity stress is one of the most common abiotic stresses that seriously damage agricultural production and endanger the sustainable food supply of the growing global human population [15,16]. More than one-third of the world’s fertile lands are affected by soil salinity, and the severity of land degradation due to salinity is expanding at the rate of 2.000–4.000 ha/day in irrigated agricultural systems of the arid and semi-arid regions [17,18]. Unfortunately, it is estimated that the impact of soil salinity will increase in the coming years and almost half of the fertile land will turn into barren lands before the 22nd century [19]. If not totally impossible, the transformation of the areas affected by salinity into a productive land is a difficult, time consuming and expensive process [20].

Salinity inflicts damage to crop plants by decreasing the osmotic potential and thus the availability of water for plant uptake, causing specific ion toxicities due to accumulation of Na^+^ and/or Cl^−^ in sensitive tissues, disturbing the essential nutrient homeostasis and also inducing oxidative stress [21]. Physiological and biochemical mechanisms contributing to salinity tolerance include changes in stomatal behavior, exclusion vs. inclusion of toxic ions, altered phytohormone metabolism, accumulation of compatible solutes for osmotic adjustment as well as induction of the antioxidative defenses. Due to the key role of oxidative stress in salinity-induced damage, the relatively higher salinity tolerance of some genotypes of a species than that of others may at least partly be attributed to a relatively higher antioxidative capacity, as it was recently shown for barley [22]. Since salinity adversely effects seed germination and seedling establishment and thereby limits the yield of crops, seed treatments with various natural bioactive compounds, including epigallocatechin-3-gallate (EGCG) [23] and γ-aminobutyric acid (GABA) [24], and synthetic agents such as silver nanoparticles (AgNPs) [25] were recently tested for their beneficial effects during early plant growth under saline conditions and demonstrated to contribute to the alleviation of salinity induced oxidative stress.

The seed germination is the first stage of plant development and very critical for the plant establishment and the yield potential. Under saline conditions, germination ratios of seeds can be severely reduced due to impaired water absorption of seeds as a result of osmotic stress, and germination may be delayed or totally blocked [26]. Low cost, efficient and eco-friendly seed treatment strategies can improve seed performance, enhance germination capacity and seedling vigor, which are crucial for healthy growth and development of plants under both control and abiotic stress conditions [27]. Seed treatments with botanical extracts, which can be a sustainable, green and promising approach in agriculture, are becoming more popular [28,29]. Botanical priming using various plant extracts, such as the *Melia azedarach* leaf extract, *Eucalyptus* leaf, ginger, garlic, mulberry, *Brassica* and *Sorghum* leaves, are reported to increase seed performance, quality and vigor, chlorophyll contents, growth and antioxidant parameters [30,31,32]. In addition to seed applications, root and foliar applications of extracts or hydrolysates obtained from various plant species can be used in crop production for their biostimulant and/or biopesticidal properties. Alfalfa (*Medicago sativa*) hydrolysates containing the plant growth regulators triacontanol and indole-3-acetic acid (IAA) were shown to stimulate growth of maize plants under saline conditions [33]. Applications of *Moringa olifera* leaf extracts to different plants, such as maize [14], pea [34] and wheat [35], were reported to enhance the abiotic stress tolerance of plants. Among commercialized plant-based extracts with pesticidal activity, neem tree (*Azdirachta indica*) extracts stand out for their effectiveness against a wide range of insect pests and their safety for people as well as beneficial insects [36].

Willows (*Salix* spp.), which are members of the Salicaceae family, are deciduous trees or shrubs. The utilization of willow tree dates back to about 6000 years ago and it is well-known as an important medicinal plant. For many years, willow barks (WB) and willow leaves (WL) were used by the ancient civilizations for the treatment of various diseases without knowing the active ingredients. Many ancient civilizations used the extracts of WB and WL due to their analgesic, antipyretic and anti-inflammatory properties [37,38]. Before the 19th century, the ingredients of willow extracts were unknown, but later studies documented the presence of many bioactive secondary compounds such as polyphenols (proanthocyanidins (Pas), phenolic acids, flavonoids, tannins, lignans), terpenoid and most importantly many salicylate compounds (salicin, saligenin, and salicylic acid) [39,40,41]. In willow, these substances play a critical role not only as a part of their defense mechanism and signaling molecule but also as therapeutic properties, especially due to salicin content. Salicin, which is the precursor of aspirin, is one of the main ingredients in WB and WL extracts [42,43]. The chemical composition and amount of salicylate compounds may show slight differences with age, seasonal, tissue, genotype, species and various environmental factors [44]. 

Salicin, which is the glucoside of salicyl alcohol (saligenin), is synthesized in plants through the phenylpropanoid pathway that begins with phenylalanine [45]. In addition, saligenin can be converted to salicin via glucosylation [46]. On the other hand, plants can synthesize salicylic acid (SA) through two pathways: isochorismate pathway (IC) and the phenylalanine ammonia-lyase (*PAL*) pathway. When SA is glucosylated, glycosylated SA can be stored in the vacuole in high amounts [47]. This glycosylated form of SA may act as a precursor of salicin [48].

The bark extract of the common plant willow has been approved by EU pesticide regulation, Regulation (EC) No 1107/2009, for agricultural applications as a basic substance with fungicidal properties [49,50]. For this purpose, the willow bark is recommended to be infused with water at 80 °C and used to control fungal diseases caused by *Taphrina deformans*, *Venturia inaequalis*, *Plasmopara viticola*, *Erysiphe necator* and *Podosphaera leucotricha* [49]. The protective mechanisms were explained by the availability of salicylic glycosides or salicylate in willow bark extracts, responsible for activating plant defense mechanisms. For agricultural applications, willow bark extracts are also recommended for accelerating root formation due to the presence of Indole 3-butyric acid (IBA) in *Salix* bark extracts [50]. In a recent study, willow bark extract is reported to speed up propagation of chrysanthemum and lavender cuttings. Although the exact mode of efficacy could not be explained, it was reported to be associated with the presence of phytohormone SA [51].

The primary aim of this study was to test the potential of WL and WB extracts obtained from local weeping willow trees (*Salix babylonica*) as novel seed treatment agents with biostimulant activity and compare the effectiveness of WL extracts with WB extracts and SA to improve the growth of maize (*Zea mays*) under both control and saline conditions. Secondly, based on the hypothesis that SA is the main bioactive component of willow extracts, we wanted to elucidate if benefits provided by willow leaf and/or bark extracts could be attributed to their SA content and also provided by exogenous SA applications. Macro- and micronutrient concentrations, protein levels, activities of antioxidative enzymes and MDA levels were measured in experimental plants.

## 2. Results

### 2.1. Chemical Characterization of Willow Extracts

Chemical analyses of willow leaf and bark extracts revealed that both extracts were rich in phenolics as well as salicylic acid (SA) and its precursors salicin and saligenin (Table 1). Willow bark (WB) extract was approximately 70% richer in phenolics than willow leaf (WL) extract. While WL extract contained five times higher concentrations of SA than WB extract, the salicin and saligenin concentrations measured in WB extract were almost two orders of magnitudes greater than those measured in WL extract. The pH of WB (4%) and WL (4%) were slightly acidic and determined as 5.31 and 5.76, respectively.

### 2.2. Germination Experiment Results

**Figure 1:**
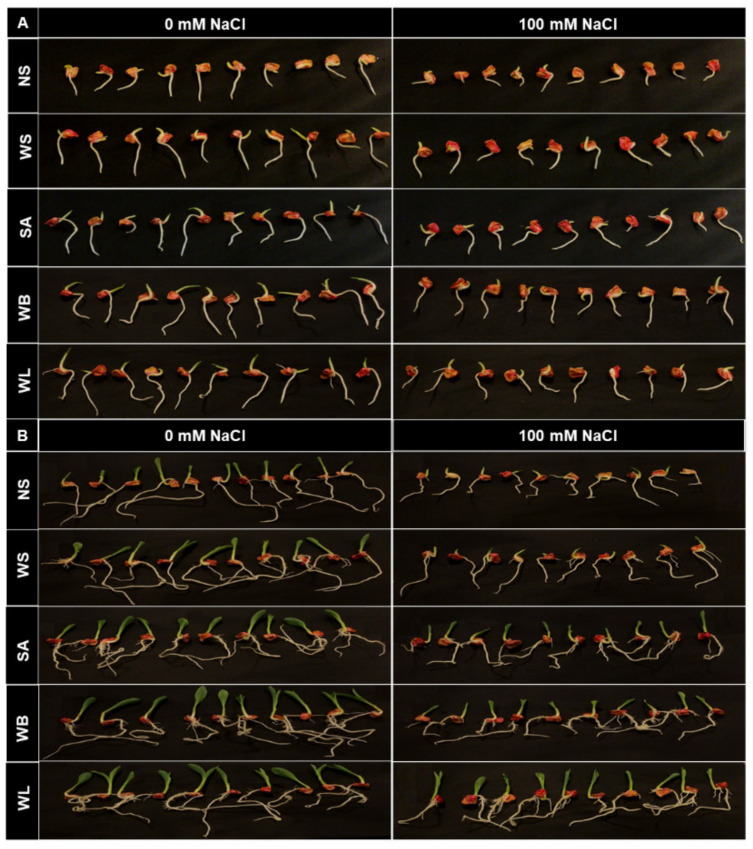
Effect of seed treatment with various agents (NS, non-soaked; WS, water-soaked; SA, salicylic acid (0.5 mM); WB, willow bark extract (2% *w*/*v*); WL, willow leaf extract (2% *w*/*v*)) on (**A**) 3-day-old and (**B**) 6-day-old maize (*Zea mays* cv. Caramelo) seedlings grown in perlite which was moistened with non-saline (0 mM NaCl) or saline (100 mM NaCl) nutrient solution under growth chamber conditions.

In the germination experiment, seed treatments of SA and the willow extracts were tested under control and saline conditions. As shown in Figure 1, salinity application adversely affected shoot and root development of maize seedlings. Under saline conditions, maize seedlings produced shorter and weaker shoots and roots at both 3 days after sowing (DAS) and 6 DAS periods. When compared with non-soaked seeds, all the seedlings which were treated with any agents (WS, SA, WB or WL) showed a better seedling performance, produced longer shoots and roots both in the presence and absence of salinity stress. Among others, both 3- and 6-day-old maize seedlings which were treated with WB and WL extracts exhibited better growth and development under the stress conditions. Visually, the differences were more obvious at 6-day-old seedlings when compared with 3-day-old ones. 

**Figure 2:**
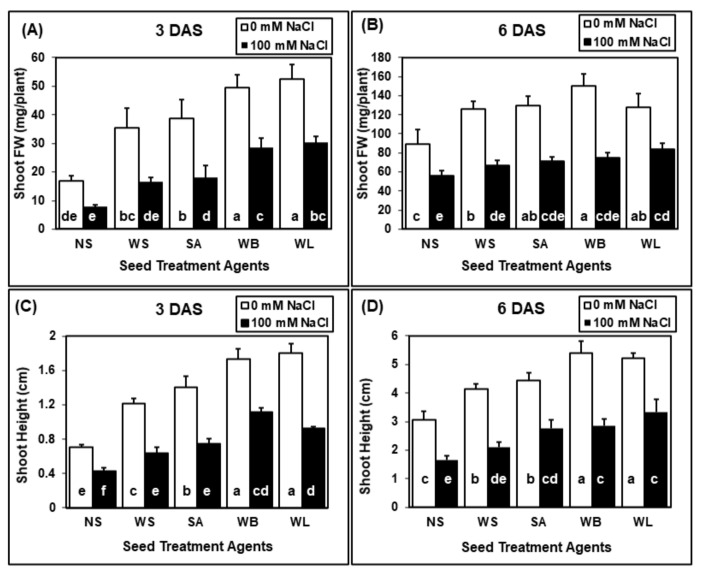
Effect of seed treatments (NS, non-soaked; WS, water-soaked; SA, salicylic acid (0.5 mM); WB, willow bark extract (2% *w*/*v*); WL, willow leaf extract (2% *w*/*v*)) on shoot fresh weight (FW) (**A**,**B**) and height (**C**,**D**) of maize (*Zea mays* cv. Caramelo) seedlings ((**A**,**C**), 3 days after sowing (DAS); (**C**,**D**), 6 DAS) grown in perlite in the absence (0 mM NaCl) or presence (100 mM NaCl) of salinity stress.

According to ANOVA results, the effects of seed treatments, salinity and their interaction on shoot FW and shoot height were significant at both 3 and 6 DAS periods (Table 2). Salinity treatment significantly reduced the shoot FW and shoot height at both harvest stages (Figure 2). The negative effects observed due to salinity stress were ameliorated with all seed treatment applications. Under saline conditions, at 3 DAS, the highest shoot fresh weight was measured in maize seedlings treated with WB or WL (Figure 2A), whereas only WL treatment caused a significant increase in shoot FW at 6 DAS (Figure 2B). The positive effects of seed soaking agents were also observed in the absence of salinity stress at both 3 and 6 DAS. During germination at both harvest stages, shoot height was reduced by approximately 45% with salinity applications (Figure 2C,D). All the seed treatment applications significantly increased the shoot biomass and height in the absence of salinity stress (Figure 2). The highest shoot FW and heights were measured in maize seedlings which were pre-treated with WB or WL extracts. At 6 DAS, the average shoot height of maize seedlings which were treated with willow extracts were 100% higher when compared with non-soaked plants (Figure 2D). 

Values are means of four independent replicates, each containing 30 seeds, and error bars represent one standard deviation. Different letters indicate significant differences according to Tukey’s HSD test (*p* < 0.05).

**Figure 3:**
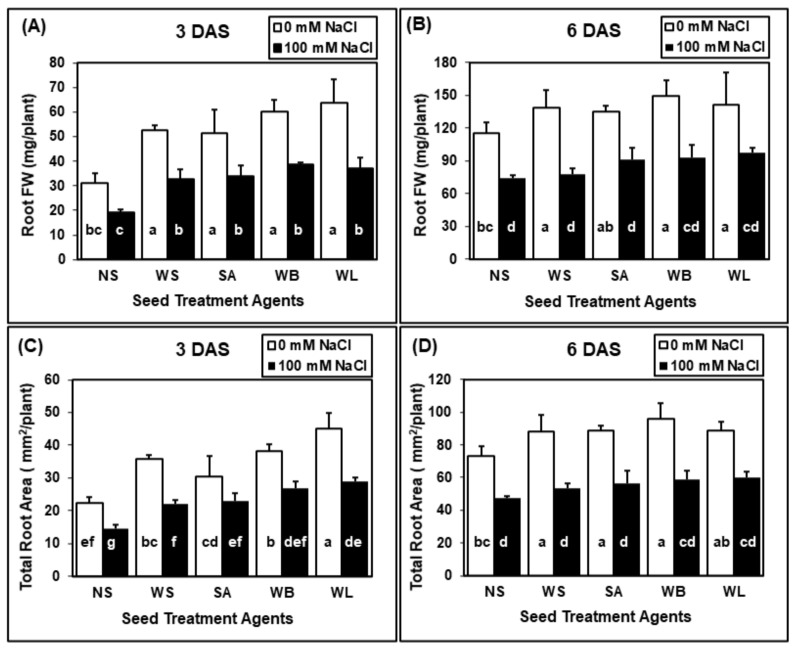
Effect of seed treatments (NS, non-soaked; WS, water-soaked; SA, salicylic acid (0.5 mM); WB, willow bark extract (2% *w*/*v*); WL, willow leaf extract (2% *w*/*v*)) on root fresh weight (FW) (**A**,**B**) and area (**C**,**D**) of maize (*Zea mays* cv. Caramelo) seedlings ((**A**,**C**), 3 days after sowing (DAS); (**C**,**D**), 6 DAS) grown in perlite in the absence (0 mM NaCl) or presence (100 mM NaCl) of salinity stress.

The root FW and area results were also significantly affected by seed treatment and salinity applications, but their interaction did not have a significant effect on these traits (Table 2). The application of water, SA and willow extracts significantly enhanced root FW of maize seedlings when compared with non-soaked treatment under non-saline conditions (Figure 3A,B). Under saline conditions, although the positive effect of seed treatments was observed in all cases, it was only significant at 3 DAS (Figure 3A,B). The highest root FWs were obtained at both harvest stages in plants grown from seeds that were treated with willow extracts. The average root FWs of the 3-day-old plants grown under both stress and control conditions were half of the root fresh weights of seedlings treated with willow extracts (Figure 3A). There was a drastic decrease in the total root area due to salinity stress (Figure 3C,D). Seed treatment agents increased the root area at both harvest times and the best results were obtained in response to willow extracts. Compared with the non-soaked seedlings, the total root area of the 3-day-old maize seedlings treated with willow leaf extract increased more than 100% in the control state and almost 100% in the case of salinity stress (Figure 3C).

Values are means of four independent replicates, each containing 30 seeds, and error bars represent one standard deviation. Different letters indicate significant differences according to Tukey’s HSD test (*p* < 0.05).

### 2.3. Soil Experiment Results

**Figure 4:**
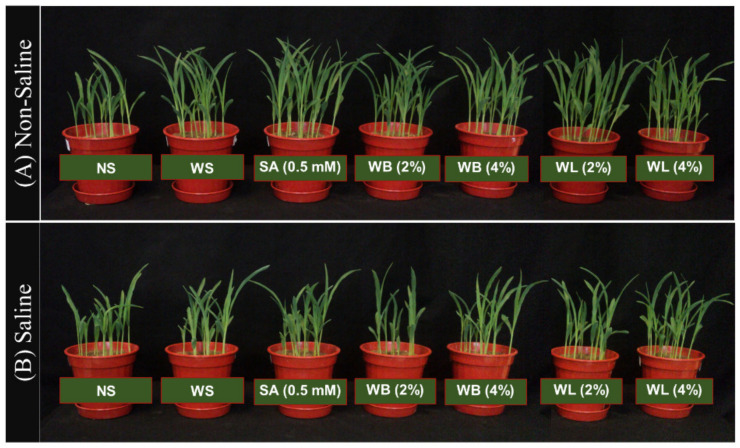
2-week-old maize (*Zea mays* cv. Caramelo) plants grown in (**A**) non-saline (EC_e_ = 3.3 dS/m) vs. (**B**) saline (EC_e_ = 7.8 dS/m) soil from seeds treated with various agents (NS, non-soaked; WS, water-soaked; SA, salicylic acid (0.5 mM); WB, willow bark extract (2% or 4% *w*/*v*); WL, willow leaf extract (2% or 4% *w*/*v*)).

**Figure 5:**
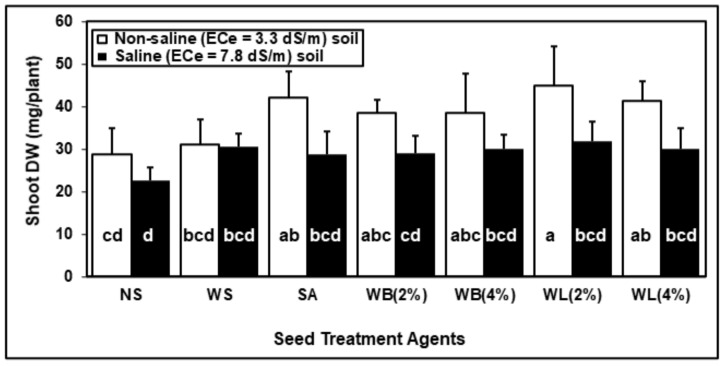
Effect of seed treatment with various agents (NS, non-soaked; WS, water-soaked; SA, salicylic acid (0.5 mM); WB, willow bark extract (2% or 4% *w*/*v*); WL, willow leaf extract (2% or 4% *w*/*v*)) on shoot dry weight (DW) of 2-week-old maize (*Zea mays* cv. Caramelo) plants grown in non-saline (EC_e_ = 3.3 dS/m) (white) vs. saline (EC_e_ = 7.8 dS/m) (black) soil.

In the soil experiment, maize seeds which were soaked by using water (WS), SA, WB and WL were grown in soil under either control or saline conditions where the soil EC*_e_* was adjusted to 7.8 dS/m for salinity treatment. According to ANOVA, the effects of seed treatments and salinity on shoot DW were significant; however, their interaction did not cause a significant effect on the reported trait (Table 3). Compared with the non-soaked plants, all the seed applications were found to have a positive effect on the growth and development of 14-day-old maize plants under both control and stress conditions (Figure 4 and Figure 5). All of the seed treatment applications enhanced the shoot biomass of maize plants under saline conditions (Figure 5). In the absence of salinity, while the effect of water soaking was minimal, applications of WL extracts caused a significant increase in shoot biomass when compared with non-soaked seeds (Figure 4 and Figure 5). Under non-saline conditions, according to shoot DW measurements, the positive effect of WL (54%) was greater than that of WB extract (38%) when compared with non-soaked plants (Figure 5). The highest shoot DW was observed as a result of seed soaking with 2% WL under both control and stress conditions (Figure 5).

Values are means of five independent pot replicates, each containing 15 seeds. Error bars represent one standard deviation. Different letters indicate significant differences according to Tukey’s HSD test (*p* < 0.05). 

ANOVA revealed a significant effect of salinity treatments on shoot Na concentration as well as the shoot concentrations of the essential macronutrients P, K, Ca and S (Table 3). Irrespective of the seed treatments, salinity increased the Na concentration approximately five-fold (Table 4). WS slightly limited the increase in Na concentration; however, SA and WB applications did not have any effect on shoot Na concentration. Interestingly, under saline conditions, Na concentrations were further increased by WL applications by approximately 15%. The shoot P concentrations were enhanced under saline conditions, and the highest P concentration was observed in non-soaked plants. All the seed treatment applications significantly reduced shoot P concentration of maize plants both in the absence and presence of salinity stress. Soil salinity reduced the shoot K concentration by 10%, irrespective of the seed treatments. The shoot Ca concentration of maize plants was reduced under saline conditions. WB applications slightly increased the Ca concentration of maize shoots in the absence of salt stress. The only macronutrient which was not significantly affected by salinity treatment was Mg. Compared with the non-soaked plant, it was observed that 4% WB and WL extracts slightly decreased shoot Mg concentration. Salt treatments caused a slight reduction in S concentrations, irrespective of the seed applications.

All seed treatment agents significantly reduced the shoot Fe concentration, particularly under saline conditions (Table 5). Salinity stress caused an increase in Zn and Mn concentrations. Zn concentration was reduced by all seed treatment applications; however, there was not a consistent effect of seed treatments on shoot Mn concentration. In contrast to other micronutrients, shoot Cu and Mo concentrations were reduced by salt treatment.

Both salinity stress and seed treatments had a significant effect on protein concentration of maize leaves, whereas their interaction did not (Table 3). In general, salinity and seed applications increased the protein concentration (Table 6). The increase caused by salinity treatment was 24% irrespective of the seed treatment. The seed treatment application enhanced protein concentrations when compared with non-soaked plants both in the absence and presence of salinity stress. When compared with WS and SA seed treatments, willow extracts caused a dramatic increase in protein levels. Under saline condition, the protein concentration was increased by 12% with WB and 27% with WL applications. 

Seed treatments caused a significant effect on the specific activities of all antioxidative enzymes including SOD, GR, APX and CAT (Table 3). Specific SOD activity was not affected by salinity but varied with seed treatments. Although WB applications did not have a consistent effect, seed treatments with WL extracts significantly reduced SOD activity under both control and saline conditions (Table 6). All the seed applications reduced specific GR activity. Application of 4% leaf extract caused the lowest GR activity under both control and saline conditions. The APX activity was reduced by all seed treatments and the greatest decrease in APX activity were measured with seed treatments of willow extracts. The specific CAT activity was significantly affected by the interaction of salinity and seed treatments. Salinity caused an increase in the specific CAT activity in the absence of seed treatments. Under saline conditions, applications of SA, WB and WL reduced the specific activity of CAT enzyme.

Values are means of five independent pot replicates, each containing 15 seeds. Error bars represent one standard deviation. Different letters indicate significant differences according to Tukey’s HSD test (*p* < 0.05). 

**Figure 6:**
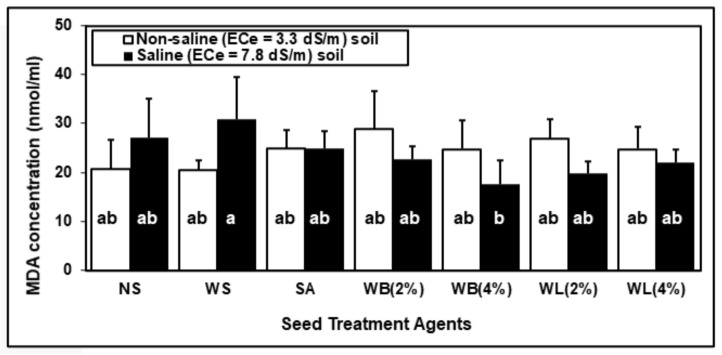
Changes in MDA concentration of 2-week-old soil-grown maize (*Zea mays* cv. Caramelo) shoots in response to salinity (non-saline: EC_e_ = 3.3 dS/m; saline: EC_e_ = 7.8 dS/m) and different seed treatments (NS, non-soaked; WS, water-soaked; SA, salicylic acid (0.5 mM); WB, willow bark extract (2% or 4% *w*/*v*); WL, willow leaf extract (2% or 4% *w*/*v*)).

Although seed treatments and salinity stress did not have a significant effect on MDA concentration, their interaction was significant (Table 3). The salinity stress increased MDA concentration in non-soaked and water-soaked groups (Figure 6). However, all seed treatment with willow tree extracts decreased the MDA concentrations in maize under the saline condition. The lowest lipid peroxidation levels were recorded for high-dose WB treatment under saline conditions.

## 3. Discussion

Seed germination is a vital stage for the lifecycle of plants and this stage is the most susceptible one to salinity stress. The germination capacity of seeds is impaired by salinity stress due to inhibition of water uptake or ionic toxicity caused by Na^+^ and Cl^−^ ions [52,53].

If seeds can germinate better under saline conditions and plantlets can tolerate salinity during seedling establishment, they have a better chance to tolerate salinity in later stages of their development [54]. For this reason, the strategies which can help seeds and seedlings overcome stress conditions are of great importance. Seed treatment agents may have a great potential to provide tolerance against various stress conditions. Through such applications, plant metabolism is stimulated and prepared for stress conditions, thus, the ability of plants to tolerate stress can be improved [55]. Usage of various plant extracts containing many bioactive substances as seed treatment agents have positive effects on plant growth parameters and improve seedlings establishment under control and stress conditions [56]. Here, seed soaking with willow extracts were shown to have a positive effect on maize seedlings during germination (Figure 1, Figure 2 and Figure 3) and early growth (Figure 4 and Figure 5).

Salicylic acid is a critical phytohormone which is involved in many critical stages of plant development. The positive effects of SA seed treatment were reported for many different plant species including wheat [57], rice [58] and maize [59]. In this study, the concentration of SA for seed soaking was 0.5 mM, corresponding to 69 mg/L SA. Although the willow extracts contained markedly lower concentrations of SA (Table 1), willow extracts were more effective than the SA treatment in enhancing various traits, including shoot FW, root FW, total root area and shoot height (Figure 1, Figure 2, Figure 3, Figure 4 and Figure 5). For 3-day-old seedlings, shoot FW was enhanced by 225% with willow extracts and by 130% with SA treatment (Figure 2). Additionally, in total root area calculations, the positive effect of SA was 43%, whereas willow extracts provided an increase by 87% (Figure 3). The positive effects observed in response to willow extracts can be due to higher concentrations of salicylate compounds other than SA itself (Table 1). Although WB had a lower SA concentration, the salicin and saligenin concentrations were much higher when compared with WL extracts (Table 1). The rich bioactive substance content of the willow extracts can be transferred to the embryo in the lag stage of germination and these active compounds including SA and other useful salicylates may increase the stress tolerance and early vigor of the seedling. Other biological compounds, including phenolics present in these crude extracts, may also contribute to the positive effects of willow extracts. Horii et al. (2007) reported that phenolic elicitors can enhance seedling vigor in tomato and pea plants [60].

During germination, the effects of seed treatments on growth parameters were reported at two different harvest times (3 and 6 DAS) (Figure 2 and Figure 3). Although the effects were more pronounced at 3 DAS, the effects were still significant at 6 DAS. For example, when compared with water application, WB seed treatment caused an increase by 50% in the shoot FW of 3-day-old seedlings, however this increase was limited to 15% for 6-day-old seedlings. When the shoot heights were compared, the heights of 3-day-old seedlings treated with willow extracts were 2.5 times the heights of non-soaked plants. However, the heights of 6-day-old maize seedlings were about 80% higher when they were treated with willow extracts. The effects of seed applications may be reduced in time but still the positive effects observed at the very early stages of a seedling’s life can be critical and may determine its survival and later performance. The results of the soil experiment also proved that the effects of seed treatments with willow extracts were still significant even at 14 DAS (Figure 4 and Figure 5).

Hydropriming, which relies on seed soaking in water and redrying prior to sowing, is known as a low cost, simple and eco-friendly seed treatment and can enhance germination and seedling establishment by improving seed hydration and metabolic responses to stress, particularly under osmotic stress conditions [61]. For various crops including sunflower (*Helianthus annuus*), green gram (*Vigna radiata*) and quinoa (*Chenopodium quinoa*) seed priming with water was shown to improve germination and seedling growth under salinity and/or drought [62,63,64]. Simply soaking seeds with water before sowing can improve crop yields by giving the germinating seeds a head start, speeding up the seedling establishment and increasing the seedling survival under stress conditions [65]. Therefore, any study investigating the potentials of seed treatment agents should ideally consider the benefits of seed soaking/priming with just water and discuss the additional benefits of the tested agents.

In the present study, significant improvements in various growth parameters were observed under both non-saline and saline conditions when maize seeds were soaked in just water (Figure 1, Figure 2 and Figure 3), which is in agreement with recent literature on seed-soaking/priming in maize [66,67,68]. Here, in terms of growth parameters including shoot and root FW, shoot height and root area, the additional benefits of the tested WL and WB extracts as seed soaking agents were most prominent during early germination and non-saline conditions (Figure 1, Figure 2, Figure 3, Figure 4 and Figure 5). However, soaking maize seeds with WB and WL extracts instead of just water significantly improved some growth parameters also under salinity stress.

Based on the data presented here, the low rate (2%) of WL extract may be the best seed treatment for enhancing various growth parameters as it is at least nearly as effective as WB applications (Figure 2, Figure 3 and Figure 5). However, for medicinal purposes, willow bark is used for different applications due to its high SA content [69,70]. Since it is much easier to collect willow leaves when compared with bark, using leaves for producing a plant-based biostimulant can be a more convenient strategy. Moreover, in some cases it was observed that the higher application rate (4%) of leaf and bark extracts increased the growth compared with non-soaked plants but, when compared with a lower willow extract dose (2%), they did not provide any extra benefit or even reduce the observed positive effects (Figure 4 and Figure 5). This can be explained by the fact that the effects of such extracts are concentration-dependent [71]. High concentrations of plant extracts may be unnecessary or even toxic for plants and have been reported to have a negative effect on plant metabolism and the secondary metabolite accumulation [72]. There is limited information in the literature suggesting negative allelopathic effects of different *Salix* species including *S. caprea* and *S. alba* on various plants; however, negative allelopathic effects of *S. babylonica* on maize or any other crop have not been reported [73,74].

Stimulation of growth and enhancement of abiotic stress tolerance by plant-based extracts is, by definition, an example of positive allelopathy, and phenolics as well as plant growth regulators (PGR) found in these extracts are among the key determinants of such effects [32,75]. Salicylic acid, which is considered the primary bioactive component in *Salix* bark extracts in traditional medicine as well as horticulture, has been extensively studied as an exogenously applied PGR to increase salinity tolerance in maize [76,77]. When applied to the growth medium, 0.5 mM SA helped maize plants tolerate salinity by decreasing the tissue sodium accumulation, improving the mineral balance, ameliorating oxidative stress, enhancing the accumulation of osmolytes and contributing to a more favorable phytohormone balance [76,78]. Foliar applications of SA to maize plants could also mitigate salinity stress by protecting the photosynthetic machinery and enhancing the antioxidant capacity [79]. Soaking maize seeds with 0.5 mM SA was reported to counteract the adverse effects of salinity treatments and improve growth parameters [77]. In the present study, there were generally no significant differences between the measured growth parameters in the water-soaking group and those in the SA-soaking group (Figure 2, Figure 3 and Figure 5), indicating that the additional benefits of WL and WB extracts could not be attributed to solely their SA contents and could not be simply mimicked by exogenous SA application.

According to the element analysis results, salinity stress decreased K and increased Na concentration in maize shoots (Table 4). Ionic toxicities, which constitute an important component of salinity stress, may also lead to reduced uptake of essential nutrients. High Na concentrations can inhibit K uptake [80]. However, it was observed that willow extracts had no positive effect on K uptake or did not decrease shoot Na concentration. This means that the protective role of willow extracts or SA cannot be explained by limited uptake or translocation of Na ions. Salinity and seed treatments also increased or reduced the concentrations of other macro- or micro-elements (Table 4 and Table 5) but none of the observed effects could explain the beneficial effects of willow extracts since none of the reported values for any mineral elements indicated a deficiency or toxicity [81]. Previously it was reported that applications of SA to the growth medium could inhibit Na and Cl uptake, enhance tissue K, Ca and Mg levels, increase K/Na ratio and stimulate the uptake of micronutrients including Fe, Mn and Cu under saline conditions [76,78]. However, in this study, where SA was used as a seed soaking agent, similar effects on mineral homeostasis were not observed (Table 4 and Table 5).

Seed treatment with SA and willow extracts increased protein concentration of maize plant grown in saline soil (Table 6). This suggests that these seed treatments enhanced nitrogen uptake. According to the literature, pre-treatment with SA may enhance nitrate reductase activity, nitrogen uptake and nitrogen use efficiency in cucumber [82], wheat [57], maize [83] and *Brassica juncea* [84]. Irrespective of salinity, seed treatments with willow extracts, particularly WL treatments, caused a significant increase in shoot protein concentrations (Table 5). The bioactive compounds found in willow extracts might enhance root N uptake or root to shoot translocation of nitrogenous compounds.

Abiotic stress, including salinity stress, leads to increasing levels of ROS in peroxisomes, chloroplasts and mitochondria [85]. It is important for the plant to control the ROS level with enzymatic (SOD, CAT, GR, APX) or non-enzymatic antioxidants (ascorbate, flavonoids carotenoids, phenolic compounds) in order to cope with oxidative stress [86]. SOD plays a role in the first step of the antioxidative defense mechanism and converts superoxide to O_2_ and hydrogen peroxide (H_2_O_2_). AP and GR involved in the ascorbate–glutathione pathway play a critical role in scavenging of ROS in chloroplast. H_2_O_2_ is also scavenged by catalase in peroxisomes [87]. According to the literature, SA treatments can increase or decrease activities of antioxidative enzymes [88,89]. According to Table 6, under saline conditions, all seed treatments except WL caused an increase in specific SOD activity. In contrast, WL applications reduced the SOD activity both in the presence or absence of salinity stress. Specific GR and APX activities decreased with all seed treatment agents and the reductions were more dramatic with willow extract treatments. The SA, as well as willow extract, treatments reduced the specific CAT activity under saline conditions. The reduced specific activities of antioxidative enzymes measured in plants grown from seeds that were treated with willow extracts may indicate a reduced ROS burden on these plants due to the direct or indirect antioxidative effects of bioactive substances found in willow extracts. High ROS levels cause oxidative damage to membranes which can be determined by increased MDA levels [90]. Seed treatment with willow extracts, especially 4% WB and 2% WL applications, diminished lipid peroxidation under salinity (Figure 6).

Previously, it was reported that seed treatments using *Moringa olifera* tree extract, which is another botanical biostimulant, enhanced the growth of maize under chilling stress and had a positive effect on root and shoot weights and heights of these plants [91]. In another study where the same tree extract was used, it was reported that seed treatments had a positive effect on various growth parameters of wheat seedlings [35].

To our knowledge, the literature on the effects of willow extracts on plants is limited to the root-enhancing and fungicidal properties of willow bark extracts [49,50,51]. Biostimulant activities of willow leaf extracts, and the applications of any willow extract as a seed treatment agent have not been reported before. As with other botanical biostimulants in the literature, willow extracts obtained from bark or leaves may serve as safe, natural and renewable inputs for sustainable agriculture. 

## 4. Materials and Methods

### 4.1. Plant Material and Growth Conditions

The maize seeds (*Zea mays* cv. Caramelo F1) which were used in this study were obtained from May Seed, Bursa, Turkey. This cultivar is a dwarf, hybrid sweet maize, which is suitable for both fresh consumption and industrial usage. Maize was selected as a model species for this study as it is one of the most important staple crops for global food security and, at the same time, it is moderately sensitive to salinity. According to the Maas-Hoffman model, which describes the relation between yield and salinity, the threshold EC_e_ for maize is 1.7 dS/m and the regression coefficient (i.e., the slope of the linear curve beyond the threshold value) is 12.9% per dS/m [92].

All the experiments were conducted in a growth chamber under controlled climatic conditions: light/dark periods, 16/8 h; temperature (light/dark), 25/20 °C; relative humidity (light/dark), 60/70.

### 4.2. Preparation of Willow Tree Extracts

For preparation of plant extracts, fresh WL and WB were collected from a mature weeping willow tree (*Salix babylonica*) from the Tuzla region of Istanbul in fall 2018. For obtaining 2% and 4% willow extracts, 15 and 30 g of WL or WB were chopped into approximately 1 cm pieces and the total volume was adjusted to 750 mL by using dH_2_O. This mixture was kept at 90 °C for 30 min and constantly stirred at 400 rpm during this process. The solution was filtered using a cheese cloth and the filtrates were centrifuged at 4000 rpm. The supernatants were collected and stored at −20 °C until further usage.

The phenolic content of willow extracts was determined by using the Folin method. The salicylate compounds of these extracts were measured by using UPLC-MS according to the method described by Blazics (2010) [93].

### 4.3. Experimental Design and Procedure

#### 4.3.1. Germination Experiment

To check the effects of willow extracts as seed treatment agents, maize seeds were soaked by using different extracts. Seed soaking applications were conducted in plastic boxes between sheets of filter paper moistened with different solutions and the seeds were kept under these conditions for 16 h at room temperature. Soaked seeds were immediately sown into plastic pots. In addition to non-soaked (NS) seeds, water-soaked (WS), salicylic acid (0.5 mM), WB extract (2%) and WL extract (2%)-soaked seeds were tested. The SA concentration was selected based on previously published studies which investigated SA as a seed treatment agent on maize [59,77,94]. In the experiment design, maize seeds were subjected to germination test in perlite where there were five seed treatment groups (including untreated controls), two salt levels (0 and 100 mM NaCl), four independent pot replicates and two different harvest times. The salinity level was determined based on preliminary tests (data not shown). Before sowing the seeds, perlite was rinsed with nutrient solution containing 0.3 mM K_2_SO_4_, 1 mM Ca(NO_3_)_2_.4H_2_O, 0.1 mM KH_2_PO_4_, 0.375 mM MgSO_4_.7H_2_O, 0.05 mM KCl, 37.5 μM Fe (in the form of FeEDTA), 1 μM H_3_BO_3_, 1 μM MnSO_4_.H_2_O, 1.5 μM ZnSO_4_.7H_2_O, 0.3 μM CuSO_4_.5H_2_0 and 0.25 μM (NH_4_)6Mo_7_O_24_.4H_2_O. For salinity applications, 100 mM NaCl was mixed with nutrient solutions. We placed 30 seeds in each plastic pot and half of the experiment was terminated at 3 days after sowing (DAS), whereas the seedlings grown in the rest of the pots were harvested at 6 DAS. The shoots and roots of maize seedlings were harvested separately, and the fresh weights were measured right after sampling. The shoot height of seedlings was measured for all plantlets. The root area of maize seedlings was determined by using all germinated seedlings, with the roots photographed on a black background and the ImageJ (2006.02.01) program used to calculate the total root area.

#### 4.3.2. Soil Experiment

Seeds, which were prepared as explained in germination experiment, were sown in pots containing 1.25 kg air-dry soil. Before sowing, each pot was fertilized with 100 ppm P (in the form of KH_2_PO_4_) and 250 ppm N (in the form of Ca(NO_3_)_2_) and mixed thoroughly. The soil was a clayey loam with an EC_e_ of 3.3 dS/m after fertilization. For salinity treatment, NaCl was added to half of the pots at the same time with fertilizers and the EC_e_ increased to 7.8 dS/m before sowing the seeds. As in the case of germination experiment, the salinity level for soil experiments was determined based on preliminary tests (data not shown). After sowing the seeds, the pots were irrigated with 50 mL distilled water daily until the experiment was terminated.

In this experiment, seeds were soaked by using seven different treatment agents (no treatment, water, SA (0.5 mM), WB (2 or 4%) and WL (2 or 4%)). The experiment had a completely randomized design with five replicates. 15 maize seeds were sown in each pot. Whole shoots of maize plants were harvested 14 DAS and washed with dH_2_O. Shoots of three plants were dipped in liquid N and stored at −80 °C until they were used for the analysis of Bradford protein, antioxidative enzyme activities and malondialdehyde (MDA). The rest of the plants were dried at 70 °C for 2 days. After determining their dry weights, they were ground, acid-digested, and analyzed for Na and mineral concentration as described below.

### 4.4. Element Analysis

Approximately 0.2 g of the dried and ground plant samples were weighed and placed in microwave digestion tubes. The samples which were treated with 2 mL of 30% hydrogen peroxide (H_2_O_2_) and 5 mL of 65% nitric acid (HNO_3_) were digested in a closed vessel microwave system (MarsExpress; CEM Corp., Matthews, NC, USA). After cooling down sufficiently, total sample volume was finalized to 20 mL by adding double-deionized water and filtered through quantitative filter papers (Macherey-Nagel, Ø125 mm, blue band). Inductively coupled plasma optical emission spectrometry (ICP-OES) (Vista-Pro Axial, Varian, Australia) was used to determine the concentrations of macro- and micronutrients as well as Na in digested plant samples. The accuracy of element analysis was checked by using certified standard reference materials obtained from the National Institute of Standards and Technology (Gaithersburg, MD, USA).

### 4.5. Extraction for Protein and Antioxidative Enzyme Assays

By mixing 50 mM KH_2_PO_4_ and 50 mM K_2_HPO_4_, a potassium phosphate (K-P) buffer with a pH of 7.6 was prepared. The extraction buffer was prepared by adding 0.1 mM EDTA Titriplex-III to this K-P buffer and kept on ice. 0.5 g of frozen maize leaf samples were homogenized in 5 mL of 50 mM K-P buffer. The homogenates were centrifuged at 4600 min^−1^ for 15 min at 4 °C, and the supernatants were transferred to microcentrifuge tubes, which were centrifuged again at 15.000 min^−1^ for 15 min at 4 °C. These supernatants were used for the determination of antioxidants enzymes (SOD, GR, AP, CAT) and protein concentrations.

### 4.6. Bradford Protein Analysis

Total protein concentration was measured by the method described by Bradford (1976) with using bovine serum albumin as standard [95]. To prepare Bradford reagent, 0.1 g Coomassie Brilliant Blue G-250 was dissolved in 50 mL ethanol and was mixed with 100 mL 85% ortho-phosphoric acid. This mixture was filtered and after filtration 100 mL glycerin was added to the reagent and the volume was brought to 1000 mL with deionized water. The reagent was kept at 4 °C for 24 h and then used for the assay. Protein standards (0, 100, 200, 400 and 800 ppm) were prepared by dissolving bovine serum albumin in K-P buffer. We added 5 mL of reagent to 0.1 mL sample (1:40 diluted) or standard and vortexed. After 10 min, the absorbance was read at 595 nm.

### 4.7. Determination of Antioxidative Enzyme Activities 

The superoxide dismutase (SOD) assay was conducted according to the method described by Giannopolitis and Ries (1977) [96]. In this method, 2.95 mL of K-P buffer was mixed with 0.5 mL of Na_2_CO_3_, L-methionine, NBT, 0.05 mL of crude sample extract (1:10 diluted) and 0.5 mL riboflavin, separately, in glass tubes. Since the chemicals used are light sensitive, they were kept under dark during preparation. After riboflavin addition, glass tubes were placed in the growth chambers and kept under light for 8 min. The measurements were performed by using a UV/Vis spectrophotometer (Cary 300 Bio, Varian, Australia) at 560 nm.

The glutathione reductase (GR) activity in shoot tissues of plants were determined by using the assay described by Carlberg and Mannervik (1985) [97]. To determine the activity of the GR enzyme, 0.7 mL of K-P buffer, 0.1 mL of Oxidized Glutathion (GSSG), 0.1 mL of 0.45 mM H_2_O_2_ and 0.1 mL of crude sample extract (1:10 diluted) were mixed and 0.1 mL of NaDPH-Na_4_ was added to this mixture. The GR activity was determined spectrophotometrically at 340 nm and absorbance values were monitored for 2 min to find the average depletion rate of NADPH-Na_4_.

The ascorbate peroxidase (APX) activity was determined according to Nakano and Asada (1981) [98]. To measure APX activity, 0.7 mL of K-P buffer was mixed with 0.1 mL of 12 mM H_2_O_2_ and 0.1 mL of crude sample extract (1:40 diluted), and then 0.1 mL of ascorbic acid (C_6_H_8_O_6_) was added. To calculate the average depletion rate of L-ascorbic acid, absorbance values at 290 nm were measured spectrophotometrically.

Catalase (CAT) activity in shoot tissues of plants were conducted by using the assay described by Chance and Maehly (1954) with slight modification [99]. The catalase activity was also determined spectrophotometrically. We mixed 0.8 mL of K-P buffer with 0.1 mL of 100 mM H_2_O_2_ and 0.1 mL of crude sample extract (1:40 diluted). The change in the absorbance of this mixture was followed for 2 min at 240 nm to calculate the average rate of H_2_O_2_ breakdown.

### 4.8. Lipid Peroxidation Assay

The frozen maize leaf samples (0.5 g) were homogenized with the help of a ball mill by using liquid N and 80% ethanol and then the homogenate was centrifuged at 3000× *g* for 10 min at 4 °C. The supernatants were transferred to microcentrifuge tubes and were used for the determination of MDA concentrations. The lipid peroxidation expressed as malondialdehyde (MDA) concentration was measured and calculated according to Hodges et al. (1999) [100].

### 4.9. Statistical Analysis

For statistical analysis, the JMP software (14.0.0) was used. The significance of the effects of the treatments and their interactions on the reported traits for each experiment was evaluated by using analysis of variance (ANOVA). Significant differences between means were determined using Tukey’s honestly significant difference (HSD) test at 5% significance.

## 5. Conclusions

It can be concluded that seed treatments with willow bark and leaf extracts could improve the seedling performance both in the presence or absence of salinity stress. Thus, seed treatments with willow extracts at optimized rates can be a promising tool for sustainable and climate-resilient crop production. Willow leaves can be as useful as willow bark for this purpose and may therefore serve as a more convenient bioeconomy resource. Considering growth promoting and stress alleviating effects as well as ease of preparation, 2% WL extract appears to be the best choice as a seed treatment agent for maize among the tested extracts and concentrations. It is thought that the biostimulant effect of willow extracts may be related to the rich bioactive substance content, including, but not limited to, salicylate compounds and phenolics. More research is required to determine if and to what extent willow extracts can be used in agriculture as natural products to replace chemical SA treatments. Results suggest that aqueous extracts of willow tissues may act as biostimulants and improve crop performance when applied to seeds, although effects may not be specific to saline conditions. Further studies are needed to verify the effectiveness of willow extracts as biostimulants on other crops under different stress conditions.

## Figures and Tables

**Table 1:** Total phenolic, salicylic acid, salicin and saligenin concentrations and pH values of weeping willow (*Salix babylonica*) leaf (WL, 4% *w*/*v*) and bark (WB, 4% *w*/*v*) extracts.

Extracts	Total Phenolics (mg GAE/L)	Salicin (mg/L)	Saligenin (mg/L)	SA (mg/L)	pH
WB (4%)	5.78	111.83	98.82	0.481	5.31
WL (4%)	3.90	2.616	2.36	2.653	5.76

**Table 2:** Significance of the effects of seed treatments (A), salinity stress (B) and their interactions on reported traits of perlite-grown maize (*Zea mays* cv. Caramelo) seedlings (3 or 6 days after sowing (DAS)) according to two-way analysis of variance (ANOVA) (α = 0.05).

Source of Variation	DF ^1^	Shoot FW		Shoot Height		Root FW		Root Area	
		3 DAS	6 DAS	3 DAS	6 DAS	3 DAS	6 DAS	3 DAS	6 DAS
Seed Treatment (A)	4	***	***	***	***	***	***	***	***
Salinity (B)	1	***	***	***	***	***	***	***	***
A × B	4	*	**	***	*	*n.s.* ^2^	*n.s.*	*	*n.s.*

^1^ DF, degree of freedom; ^2^ *n.s.*, not significant; * 0.01 < *p* < 0.05; ** 0.001 < *p* < 0.01; *** *p* < 0.001.

**Table 3:** Significance of the effects of seed treatments (A), salinity stress (B) and their interactions on reported traits of soil-grown maize (*Zea mays* cv. Caramelo) seedlings according to two-way analysis of variance (ANOVA) (α = 0.05).

**Source of Variation**	**DF ^1^**	**Shoot DW**	**Na**	**P**	**K**	**Ca**	**Mg**	**S**	**Fe**	**Zn**
Seed Treatment (A)	6	***	*n.s.* ^2^	***	**	*n.s.*	***	*	**	***
Salinity (B)	1	***	***	*	***	**	*n.s.*	***	*n.s.*	***
A × B	6	*n.s.*	*n.s.*	*n.s.*	*n.s.*	*n.s.*	*n.s.*	*n.s.*	*n.s.*	*n.s.*
**Source of Variation**	**DF**	**Mn**	**Cu**	**Mo**	**Protein**	**SOD**	**GR**	**APX**	**CAT**	**MDA**
Seed Treatment (A)	6	*	**	*n.s.*	**	**	***	***	*	*n.s.*
Salinity (B)	1	***	***	***	***	*n.s.*	*n.s.*	*n.s.*	*	*n.s.*
A × B	6	*n.s.*	*n.s.*	*n.s.*	*n.s.*	*n.s.*	*n.s.*	*n.s.*	***	**

^1^ DF, degree of freedom; ^2^ *n.s*., not significant; * 0.01 < *p* < 0.05; ** 0.001 < *p* < 0.01; *** *p* < 0.001.

**Table 4:** Changes in shoot Na and macronutrient (P, K, Ca, Mg, S) concentrations of 2-week-old soil-grown maize (*Zea mays* cv. Caramelo) plants in response to salinity (EC_e_ = 3.3 dS/m; EC_e_ = 7.8 dS/m) and different seed treatments (NS, non-soaked; WS, water-soaked; SA, salicylic acid (0.5 mM); WB, willow bark extract (2% or 4% *w*/*v*); WL, willow leaf extract (2% or 4% *w*/*v*)).

Seed Treatments	Soil EC_e_ (dS/m)	Na (%)			P (%)			K (%)			Ca (%)			Mg (%)			S (%)		
NS	3.3	0.06	±	0.02	0.84	±	0.07	3.9	±	0.1	0.66	±	0.06	0.47	±	0.02	0.42	±	0.01
	7.8	0.38	±	0.08	0.91	±	0.04	3.5	±	0.1	0.61	±	0.05	0.47	±	0.02	0.37	±	0.02
WS	3.3	0.06	±	0.01	0.77	±	0.09	3.8	±	0.1	0.67	±	0.03	0.46	±	0.02	0.41	±	0.01
	7.8	0.33	±	0.07	0.76	±	0.07	3.6	±	0.1	0.59	±	0.03	0.46	±	0.01	0.35	±	0.01
SA	3.3	0.05	±	0.01	0.78	±	0.09	4.0	±	0.1	0.66	±	0.06	0.47	±	0.02	0.40	±	0.01
	7.8	0.39	±	0.07	0.80	±	0.06	3.6	±	0.1	0.63	±	0.02	0.46	±	0.02	0.35	±	0.01
WB (2%)	3.3	0.05	±	0.01	0.73	±	0.06	3.8	±	0.1	0.70	±	0.09	0.45	±	0.02	0.40	±	0.02
	7.8	0.39	±	0.05	0.79	±	0.03	3.7	±	0.2	0.65	±	0.06	0.46	±	0.01	0.36	±	0.02
WB (4%)	3.3	0.05	±	0.01	0.73	±	0.08	3.8	±	0.3	0.74	±	0.10	0.45	±	0.02	0.40	±	0.01
	7.8	0.39	±	0.04	0.74	±	0.07	3.5	±	0.1	0.64	±	0.03	0.43	±	0.04	0.35	±	0.02
WL (2%)	3.3	0.04	±	0.01	0.70	±	0.06	3.8	±	0.2	0.65	±	0.03	0.45	±	0.03	0.41	±	0.02
	7.8	0.44	±	0.04	0.75	±	0.04	3.5	±	0.1	0.69	±	0.04	0.45	±	0.01	0.35	±	0.01
WL (4%)	3.3	0.06	±	0.01	0.72	±	0.05	3.7	±	0.1	0.66	±	0.05	0.42	±	0.02	0.39	±	0.02
	7.8	0.43	±	0.02	0.75	±	0.07	3.4	±	0.2	0.66	±	0.05	0.43	±	0.02	0.35	±	0.01

Na, HSD_0.05_ (ST; EC_e_; ST × EC_e_) = (*n.s*.; 0.02; *n.s.*); P, HSD_0.05_ (ST; EC_e_; ST × EC_e_) = (0.09; 0.03; *n.s.*); K, HSD_0.05_ (ST; EC_e_; ST × EC_e_) = (0.2; 0.07; *n.s.*); Ca, HSD_0.05_ (ST; EC_e_; ST × EC_e_) = (*n.s.*; 0.03; *n.s.*); Mg, HSD_0.05_ (ST; EC_e_; ST × EC_e_) = (0.03; *n.s.*; *n.s.*); S, HSD_0.05_ (ST; EC_e_; ST × EC_e_) = (0.02; 0.007; *n.s.*).

**Table 5:** Changes in micronutrient (Fe, Zn, Mn, Cu, Mo) concentrations of 2-week-old soil-grown maize (*Zea mays* cv. Caramelo) shoots in response to salinity (non-saline, EC_e_ = 3.3 dS/m; saline, EC_e_ = 7.8 dS/m) and different seed treatments (NS, non-soaked; WS, water-soaked; SA, salicylic acid (0.5 mM); WB, willow bark extract (2% or 4% *w*/*v*); WL, willow leaf extract (2% or 4% *w*/*v*)).

Seed Treatments	Soil EC_e_ (dS/m)	Fe (mg.kg^−1^)			Zn (mg.kg^−1^)			Mn (mg.kg^−1^)			Cu (mg.kg^−1^)			Mo (mg.kg^−1^)		
NS	3.3	150	±	20	80	±	9	101	±	7	16.5	±	0.5	5.8	±	1.3
	7.8	171	±	28	95	±	11	121	±	13	15.8	±	0.8	2.1	±	0.3
WS	3.3	147	±	28	67	±	5	93	±	11	16.0	±	0.6	6.4	±	1.8
	7.8	137	±	12	77	±	10	100	±	7	14.4	±	0.4	2.5	±	0.5
SA	3.3	136	±	8	65	±	6	92	±	10	15.3	±	0.4	5.3	±	1.3
	7.8	136	±	10	76	±	5	110	±	6	14.4	±	0.7	2.2	±	0.4
WB (2%)	3.3	135	±	8	63	±	5	85	±	7	15.3	±	0.7	6.5	±	1.7
	7.8	130	±	12	75	±	5	110	±	10	14.5	±	0.8	2.3	±	0.1
WB (4%)	3.3	148	±	42	66	±	8	101	±	15	15.1	±	1.1	5.2	±	2.2
	7.8	119	±	7	69	±	6	102	±	16	14.6	±	2.0	2.3	±	0.3
WL (2%)	3.3	138	±	6	58	±	5	94	±	6	14.8	±	0.4	5.3	±	1.2
	7.8	129	±	13	71	±	4	115	±	9	14.6	±	1.9	2.3	±	0.4
WL (4%)	3.3	139	±	8	62	±	5	104	±	9	15.1	±	0.3	4.0	±	0.4
	7.8	127	±	4	72	±	7	115	±	18	13.7	±	0.4	2.2	±	0.4

Fe, HSD_0.05_ (ST; EC_e_; ST × EC_e_) = (24; *n.s.*; *n.s.*); Zn, HSD_0.05_ (ST; EC_e_; ST × EC_e_) = (9; 3; *n.s.*); Mn, HSD_0.05_ (ST; EC_e_; ST × EC_e_) = (15; 5; *n.s.*); Cu, HSD_0.05_ (ST; EC_e_; ST × EC_e_) = (1.3; 0.5; *n.s.*); Mo, HSD_0.05_ (ST; EC_e_; ST × EC_e_) = (*n.s.*; 0.5; *n.s.*).

**Table 6:** Changes in protein concentrations and specific activity of antioxidative enzymes (SOD, GR, APX, CAT) of 2-week-old soil-grown maize (*Zea mays* cv. Caramelo) shoots in response to salinity (non-saline, EC_e_ = 3.3 dS/m; saline, EC_e_ = 7.8 dS/m) and different seed treatments (NS, non-soaked; WS, water-soaked; SA, salicylic acid (0.5 mM); WB, willow bark extract (2% or 4% *w*/*v*); WL, willow leaf extract (2% or 4% *w*/*v*)).

Seed Treatments	Soil Ec_e_ (dS/m)	Protein Concentration			SOD			GR			APX			CAT		
		(mg.g^−1^ FW)			(U mg^−1^ Prt)			(-nmol [NADPH] mg^−1^ Prt min^−1^)			(-µmol H_2_O_2_ mg^−1^ Prt min^−1^)					
NS	3.3	6.3	±	0.8	28	±	6	76	±	23	2.2	±	0.4	47	±	8
	7.8	7.8	±	0.6	25	±	5	74	±	10	1.9	±	0.4	72	±	9
WS	3.3	6.8	±	2.2	30	±	11	58	±	12	1.7	±	0.2	55	±	6
	7.8	8.8	±	1.1	28	±	1	66	±	19	1.6	±	0.3	75	±	9
SA	3.3	7.5	±	1.2	30	±	6	68	±	13	1.4	±	0.5	73	±	14
	7.8	7.1	±	2.4	33	±	5	58	±	12	1.6	±	0.2	65	±	8
WB (2%)	3.3	7.8	±	1.0	21	±	9	51	±	5	1.7	±	0.3	65	±	9
	7.8	9.0	±	0.9	27	±	3	55	±	12	1.1	±	0.3	66	±	12
WB (4%)	3.3	7.8	±	1.4	29	±	5	48	±	8	1.2	±	0.2	76	±	6
	7.8	8.7	±	3.0	29	±	8	49	±	4	1.3	±	0.2	56	±	8
WL (2%)	3.3	8.4	±	2.2	23	±	5	43	±	5	1.3	±	0.3	54	±	13
	7.8	9.8	±	0.7	24	±	2	51	±	5	1.2	±	0.3	57	±	11
WL (4%)	3.3	8.8	±	1.4	21	±	3	42	±	12	1.3	±	0.1	50	±	7
	7.8	10.0	±	0.9	21	±	3	42	±	2	1.1	±	0.3	62	±	9

Protein Concentration, HSD_0.05_ (ST; EC_e_; ST × EC_e_) = (1.8; 0.6; 2.9); SOD, HSD_0.05_ (ST; EC_e_; ST × EC_e_) = (8.2; *n.s.*; *n.s.*); GR, HSD_0.05_ (ST; EC_e_; ST × EC_e_) = (16.5; *n.s*.; *n.s.*); APX, HSD_0.05_ (ST; EC_e_; ST × EC_e_) = (0.4; *n.s*.; *n.s.*); CAT, HSD_0.05_ (ST; EC_e_; ST × EC_e_) = (13.6; 4.8; 22.8).

## Data Availability

The data presented in this study are available on request from the corresponding author.
